# How Soluble GARP Enhances TGFβ Activation

**DOI:** 10.1371/journal.pone.0153290

**Published:** 2016-04-07

**Authors:** Sven Fridrich, Susanne A. Hahn, Marion Linzmaier, Matthias Felten, Jenny Zwarg, Volker Lennerz, Andrea Tuettenberg, Walter Stöcker

**Affiliations:** 1 Cell and Matrix Biology, Institute of Zoology, JGU Mainz, Mainz, Germany; 2 University Hospital Mainz, Dermatology, Mainz, Germany; 3 University Hospital Mainz, 3rd medical center, Mainz, Germany; Jackson Laboratory, UNITED STATES

## Abstract

GARP (glycoprotein A repetitions predominant) is a cell surface receptor on regulatory T-lymphocytes, platelets, hepatic stellate cells and certain cancer cells. Its described function is the binding and accommodation of latent TGFβ (transforming growth factor), before the activation and release of the mature cytokine. For regulatory T cells it was shown that a knockdown of GARP or a treatment with blocking antibodies dramatically decreases their immune suppressive capacity. This confirms a fundamental role of GARP in the basic function of regulatory T cells. Prerequisites postulated for physiological GARP function include membrane anchorage of GARP, disulfide bridges between the propeptide of TGFβ and GARP and connection of this propeptide to α_v_β_6_ or α_v_β_8_ integrins of target cells during mechanical TGFβ release. Other studies indicate the existence of soluble GARP complexes and a functionality of soluble GARP alone. In order to clarify the underlying molecular mechanism, we expressed and purified recombinant TGFβ and a soluble variant of GARP. Surprisingly, soluble GARP and TGFβ formed stable non-covalent complexes in addition to disulfide-coupled complexes, depending on the redox conditions of the microenvironment. We also show that soluble GARP alone and the two variants of complexes mediate different levels of TGFβ activity. TGFβ activation is enhanced by the non-covalent GARP-TGFβ complex already at low (nanomolar) concentrations, at which GARP alone does not show any effect. This supports the idea of soluble GARP acting as immune modulator *in vivo*.

## Introduction

The pleiotropic cytokine TGFβ1 (transforming growth factor) is found throughout the metazoan kingdom and fulfills multiple functions in development and tissue differentiation [1]. TGFβ1 deficient mice die shortly after birth due to multi-organ inflammation [2], which points out its homeostatic role as a strongly immunosuppressive agent [3]. TGFβ1 is translated as inactive pro-TGFβ1, it forms homo dimers and gets furin-cleaved between the propeptide and the mature chain in the Golgi [4]. In the resulting ‘latent TGFβ1’ or ‘small latent complex’ (SLC) the homo dimeric cytokine is non-covalently bound to its own propeptide termed ‘latency associated peptide’ (LAP). In most cells, the latent TGFβ1 is carried by ‘latent TGFβ binding proteins’ (LTBPs). This so-called ‘large latent complex’ is secreted and becomes tethered to components of the extracellular matrix such as fibrillin, fibronectin and fibulin prior to activation [4].

**Fig 1:**
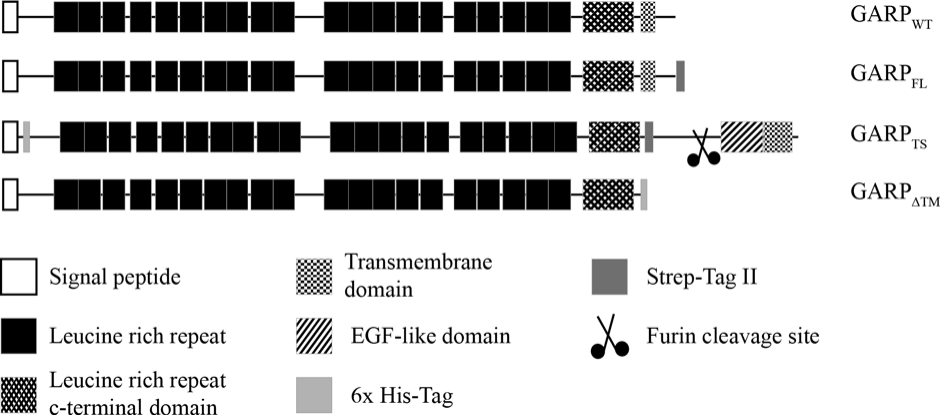
Domain structure of GARP and its recombinant variants. Schematic representation of human GARP and its recombinant variants used in this study. GARP consists of a signal peptide, 20 leucine rich repeats, a leucine rich repeat C-terminal flanking domain and a transmembrane region. For the construct GARP_FL_ a Strep-tag was added at the intracellular C-terminus. Instead of the original GARP transmembrane region, the construct GARP_TS_ possesses the transmembrane region of the protease meprin α and additionally its extracellular EGF-like and inserted domain. This construct was cleaved by furin in the trans-Golgi network and secreted into the extracellular space. For purification and detection a Strep-tag was inserted between the extracellular part of GARP and the meprin α part and a His-tag between the signal peptide and the mature chain. GARP_ΔTM_ lacks the complete transmembrane region of GARP, but contains a His-tag instead at the C-terminus of the extracellular part.

A completely different latent TGFβ1 binding protein termed GARP (glycoprotein A repetitions predominant) has been discovered on platelets [5] and on activated regulatory T-lymphocytes (Treg) [6] and, most recently, on hepatic stellate cells [7]. GARP is a glycosylated type 1 membrane protein consisting of 20 leucine rich repeats (LRR), a leucine-rich repeat C-terminal flanking domain and a membrane spanning domain (Fig 1) [8]. Thus, it is a typical member of the LRR containing proteins together with TOLL-like cell surface receptors or extracellular matrix proteins like biglycan and decorin, which all share a horseshoe shape conformation [9,10]. The latent TGFβ1 complex is bound to the extracellular part of GARP at the cell surface [11,12].

Several lines of evidence suggest GARP to be intimately involved in the immunosuppressive function of Treg and in the maintenance of self-tolerance. A knockdown or dysfunction of FOXP3, a major transcription factor of Treg, resulted in systemic autoimmune disease in mice and humans [13]. GARP expression correlates with the TGFβ1 mediated immunosuppression, since TGFβ1 null mice show the same phenotype as the FOXP3 knockout [2]. A knockdown of GARP with shRNA in *ex vivo* Treg reduced their suppressive capacity by half [14]. Furthermore, pancreas homing Treg of NOD mice (non-obese diabetic), which develop spontaneous diabetes type I, exhibited a strongly reduced GARP expression [15], but could be rescued by TGFβ1 overexpression in the pancreas [16]. Moreover, Treg were observed to be strongly expanded in HIV patients [17], and in feline immunodeficiency virus infected cats, GARP is specifically up-regulated compared to non-infected animals [18]. In this setting, virtually any suppressive actions of Treg could be diminished by using blocking antibodies against GARP or TGFβ1, respectively [18]. In certain cancers, such as hepatocellular carcinomas, Treg express significantly more GARP, which correlates with elevated TGFβ1 blood levels [19].

Although the immune suppressive role of TGFβ1 has been known for long, there are still open questions concerning its mode of presentation, activation and action as a paracrine and autocrine cytokine in the immune system. It had been shown previously for the large latent TGFβ1-LTBP1 complex that LTBP1 forms disulfide bonds to the LAP before it is translocated to the cell surface [20]. More recently, the same was shown for the latent TGFβ1-GARP complex [11]. For the release of mature TGFβ1 from the large latent complex several mechanisms have been suggested, including proteolysis by BMP1, MT1-MMP, MMP2, MMP9 and Plasmin and/or tensile forces by α_v_β_6_ and/or α_v_β_8_ integrins of neighboring target cells [4]. It has been proposed that membrane tethering, disulfide bonding to GARP and the presence of intact RGD-motifs are prerequisites for effective TGFβ1 signaling [21]. However, latent TGFβ1 is produced by activated T cells not only as a cell surface bound cytokine, but also as a soluble complex, which needs to be activated by a hitherto unknown release mechanism [22]. In addition, also soluble latent TGFβ1-GARP complexes have been observed, possibly due to proteolytic shedding [23]. The mechanism of this shedding process, its regulation and the activation of latent TGFβ1 from these complexes are not known yet. However, application of high doses of soluble GARP to naïve T cells induced expression of TGFβ1 and FoxP3, which converts them into induced Treg (iTreg), and these effects could be diminished by the application of TGFβ receptor blocking antibodies [24]. This can be interpreted as indirect evidence for an interaction of soluble GARP and soluble latent TGFβ in the extracellular space.

In order to study the underlying molecular mechanism of this interaction, we produced a biologically fully active soluble GARP-variant, which was translated with the membrane anchor of the human metalloproteinase meprin α, to introduce a furin cleavage site causing secretion into the extracellular space. This soluble GARP bound pro-TGFβ1 as well as latent TGFβ1 and it enhanced the conversion of the latent TGFβ1 to its active form. Moreover, two different ways of GARP-TGFβ1 interaction could be observed, either covalent or non-covalent. These two species of GARP-TGFβ complexes behave differently regarding the activability of bound TGFβ, which would explain the observations reported by Wang et al. (2012) [21] and Hahn et al. (2013) [24].

## Material and Methods

### Material

The GARP cDNA clone IRATp970C0699D and the TGFβ cDNA clone IRATp970G0838D were purchased at imaGenes GmbH (Berlin, Germany), Meprin α cDNA was a kind gift of Prof. Dr. Erwin Sterchi (University of Berne, Switzerland). Primers were purchased at Biomers.net GmbH (Ulm, Germany) and restriction enzymes and PCR reagents were supplied by NEB (Frankfurt/Main, Germany). Cell culture reagents, HEK 293H (human embryonic kidney) cells, pIRES-neo2 and pFastBac1 expression vectors were ordered from Invitrogen (Darmstadt, Germany). The expression vector pDsRed-Monomer-HygN1 was a kind gift of Dr. Oliver Schilling (Albert-Ludwigs-University, Freiburg, Germany). SF9 and Hi5 Insect cells and Mv1Lu mink cells were obtained from Friedrich-Löffler Institute (Greifswald, Germany). All other reagents were from Applichem GmbH (Darmstadt, Germany) or Carl-Roth GmbH & Co. GK (Karlsruhe, Germany). DNA constructs were sequenced at Starseq (Mainz, Germany).

### Attachment of an N-terminal 6x His-tag to GARP

An N-terminal 6x His-tag was inserted between the signal peptide and the mature chain of the GARP receptor. For this purpose, primers were used binding in the 5’UTR region of the GARP cDNA containing a *Nhe*I site (GCTAGCAGCTGAGCGGCCTGCTCCTCCTCG; primer 1) and at the link between the signal peptide and the mature chain. This primer adds the codons for five histidine residues after H20 and also an *Xho*I restriction site (CTTACAGGGCACTTTCTCGAGTTG ATGGTGATGGTGATGGTGTTGTGCAGCCAG; primer 2) resulting in the insertion of a leucine after Q26 and the point mutation of D28→E28. The second PCR fragment was amplified using the reverse complementary version of primer 2 and a primer binding at the 3’UTR region of the GARP cDNA, which contains a natural occurring *BamH*I site (ATTTGGAGACCAGAGTTCTGGGATCCC GGATCACTG; primer 3). Both PCR products were cloned into the pGEM-T Vector (Promega; Mannheim, Germany) and fused using the *Xho*I site. For expression, the recombinant cDNA was cloned into the pIRES-AcGFP Vektor (BD Biosciences; Heidelberg, Germany).

### Tail switch of GARP and Meprin α

In order to switch the transmembrane region of GARP, a cDNA fragment was amplified using primer 1 (see above) and a primer binding at the 3’-end of the segment encoding the extracellular part of GARP up to N632. This primer also contains the sequence coding for a Strep-tag II, which serves as linker and enables the fusion with the meprin α tail via an internal *BstB*I site (TTTTTCGAACTGCG GGTGGCTCCAGTTGATGTTCTTCAGTCCCCCCTT; primer 4). The pGEM-T containing the GARP_FL_-His sequence was used as a template to keep the N-terminal His-tag. The meprin α tail was amplified using a primer encoding the Strep-tag II sequence together with a 5’ *BstB*I site and the coding sequence starting at P650 (TGGAGCCACCCGCAGTTCGAAAAACCCTCTAAAGGCAAA AGACTGAGC; primer 5). The reverse primer binds at the 3’UTR region (CTCGAGGAAAGTT AAGGCCTGCATGGAGGA; primer 6). Both fragments were amplified and cloned in the pGEM-T Vector as described above. The *BstB*I site was used to join both fragments and the whole construct was cloned in expression vectors for mammalian and insect cells. The resulting construct was named GARP_TS_. For protein expression, mammalian cells were transfected with the pIRES-neo2 construct. In case of insect cells we used the baculovirus system containing the expression vector pFastBac1.

### Cloning of a GARP-ΔTM mutant

The transmembrane region of the GARP receptor was deleted to investigate whether the membrane anchor is necessary for the expression of a soluble GARP construct. Therefore a PCR was carried out with primer 1 and a primer similar to primer 4 (see above) but not containing a Strep-tag II but a 6x His-tag, a *BamH*I site and a stop codon (GTGGATCCTAGTGATGGTGATGGTGATGGTTGATGT TCTTCAGTC; primer 7). This PCR product was amplified using the wild-type GARP sequence and cloned in the expression vectors pIRES-neo2 and pFastBac1 as described above.

### Cloning of full-length GARP with a C-terminal Strep-tag II

For purification and for comparison the recombinant GARP constructs with the wild-type GARP receptor, a Strep-Tag II was added C-terminally to the intracellular cytoplasmic tail. Therefore a PCR was performed using primer 1 (see above) and a primer containing the Strep-tag II sequence, stop codon and a *BamH*I restriction site (TCGGATCCTATTTTTCGAACTGCGGGTGGCTCCAGGCTT TATACTGTTGGTTAAACTTC; primer 8). As template the wild-type sequence was used and the PCR product was first cloned in the pGEM vector and afterwards in the expression vector pIRES-neo2. This construct was named GARP_FL_.

### Cloning of TGFβ with an N-terminal Strep-tag II

In order to express and purify TGFβ in sufficient quantity for biochemical analysis, a Strep-tag II was inserted between the cDNA sequences encoding the signal peptide and the LAP of TGFβ. For this purpose a PCR was performed resulting in a segment coding for an FseI restriction site at the 5’-end and the Strep-tag at the border between signal peptide and the LAP. The primers used were: GGCCGGCCGGCCGCGGGACTATCCTGGAGCCACCCGCAGTTCGAAAAAACCTGCAAGACT and GAGAATTCTAGCTGCACTTGCAGGAGCGCACGATCATGTTG. Because of the FseI site this PCR fragment could be ligated back to the natural signal peptide-coding region and cloned afterwards in the expression vector for the baculovirus expression system pFastBac1.

### Recombinant protein expression in HEK 293H cells

HEK 293H cells (Invitrogen; Darmstadt, Germany) were transfected in 6-well plates in DMEM, supplemented with 10% heat inactivated FCS using Xtreme Gene HP (Roche; Grenzach-Wyhlen, Germany) following the producer’s instructions. After 48 h the transfection medium was removed, cells were washed twice with PBS and incubated for another 48 h in FCS free DMEM supplemented with 1X non-essential amino acids (NEAA). After 48 h the supernatant was collected. The cells were washed twice with PBS and lysed using RIPA buffer (50 mM Tris/HCl, pH 7.4, 150 mM NaCl, 1% (v/v) Triton-X 100, 1% (w/v) sodium deoxycholate; 0.1% (w/v) SDS, 1 mM Na_2_EDTA). Supernatants and cell lysates were stored at -20°C for further analysis.

### Expression and purification of recombinant GARP_TS_ and TGFβ in Hi5 insect cells

Baculoviruses were prepared in *Spodoptera frugiperda* (SF9) cells and BTI-TN-5B1-4 (Hi5) cells (from *Trichoplusia ni*), were infected following the producer’s instructions. Briefly, Hi5 insect cells were cultivated in serum free Express Five medium (Thermo Scientific; Schwerte, Germany) at 25°C. Cells were grown in spinner flasks and previous of the infection transferred into Fernbach flasks. The Infection with recombinant baculovirus (MOI 1) was then performed at a cell density of 1.8–2.2. The expression was stopped 96 h post infection. Conditioned medium was buffered by 20 mM NaOAc, pH 5.5 and was centrifuged for 20 min at 5000 x g at 4°C. Proteins of the cleared conditioned media were bound to Affi-Gel Blue (Biorad; Munich, Germany) in a batch process at 4°C over night. The Affi-Gel Blue was loaded onto a column and washed first with binding buffer (20 mM NaOAc, pH 5.5, 150 mM NaCl) and then with washing buffer (20 mM Tris/HCl pH 8.0; 300 mM NaCl). The elution was initiated with 20 mM Tris/HCl pH 8.0; 2 M NaCl. Eluates were dialyzed against 50 mM Tris/HCl, pH 8.0; 100 mM NaCl; (10 mM imidazole, only GARP_TS_) and loaded on a Ni-NTA Sepharose (Qiagen; Hilden, Germany) for GARP_TS_ or on a Strep-Tactin (IBA Lifesciences GmbH; Göttingen, Germany) column for TGFβ. For GARP_TS_, the column was washed with buffer containing, 50 mM imidazole before elution by 50 mM Tris/HCl, pH 8.0, 100 mM NaCl, 100 mM imidazole. TGFβ was washed on the Strep-Tactin column with binding buffer and eluted with binding buffer containing 2.5 mM D-desthiobiotin. Proteins were analyzed by polyacrylamide electrophoresis and immunoblotting. For gel electrophoresis the Biorad Mini-Protean system was used. Samples were applied to 10% polyacrylamide gels and separated at 120 V in 192 mM glycine, 0.02% (w/v) SDS, 25 mM Tris/HCl pH 8.3 as running buffer. For immunoblotting the proteins were transferred onto a PVDF membrane using 40 mM glycine, 20% (v/v) ethanol, 25 mM Tris/HCl pH 8.0 as cathode buffer, 20% (v/v) ethanol, 300 mM Tris/HCl pH 10.4 as anode buffer and a constant voltage of 20 V for 2 h. For immunodetection anti-Strep or anti-His antibodies (1:1000 in 3% (w/v) TBS) from Qiagen (Hilden, Germany) and horseradish peroxidase coupled anti-mouse-IgG antibody (Dianova; Berlin, Germany) (1:10000 in 10% (w/v) non-fat dried milkpowder) were used, respectively. For chemiluminescent detection the Biorad (Hilden, Germany) Clarity Western ECL solution was used.

### Far-UV CD-Spectroscopy

Far UV Spectroscopy was performed in a Jasco J-810 CD-spectrometer using a 1 mm Hellma CD-cuvette at 25°C. The spectra were recorded from 300 nm to 185 nm with a scanning speed of 1 nm/s. Each sample was measured five times and presented as relative mean ellipticity (deg · cm^2^ · dmol^-1^). Purified recombinant TGFβ and GARP_TS_ were dialyzed in 100 mM NaF and 50 mM NaH_2_PO_4_ pH 7.4 and concentrated to 0.8 mg/ml and 1.2 mg/ml, respectively.

### TGFβ pull-down assay

Binding of TGFβ to GARP_TS_ was assessed in a pull-down assay with Ni-NTA magnetic beads (Qiagen; Hilden, Germany). This was possible since the recombinant GARP_TS_ contains a 6x His-tag at its N-terminus. GARP_TS_ and TGFβ were incubated in 50 mM Tris/HCl, pH 8.0, 100 mM NaCl over night at RT or in the same buffer containing 2 mM cysteine and 0.5 mM oxidized glutathione system for disulfide bonding. Ni-NTA magnetic beads equilibrated in the same buffer were added and incubated at 4°C for 4 hours. Then the beads were separated from the supernatant magnetically and washed with 50 mM Tris/HCl, pH 8.0, 100 mM NaCl, 25 mM imidazole. Proteins were eluted with 20 mM NaOAc, pH 4.5, 2 M urea, 150 mM NaCl.

### Mv1Lu cell proliferation assay

To examine the enhancement of TGFβ activity by GARP_TS_, the proliferation of Mv1Lu cells was determined as described [25]. Briefly, 2.5 x 10^4^ Mv1Lu cells per well were seeded on a 96 well plate in DMEM supplemented with 1% FBS, 1x NEAA and 100 U/ml penicillin, 100 μg/ml streptomycin. TGFβ, GARP_TS_ or both respectively were added after cells have attached to the well, usually after 2 hours after seeding. The readout was performed 48 hours after cytokine application. Cells were fixed with 2% w/v paraformaldehyde in PBS and stained with methylene blue. After 5 washes with 10 mM Na_2_B_4_O_7_—buffer, pH 8.5, cell bound methylene blue was resolved in a 1:1 mixture of 100 mM HCl and ethanol. Absorption was measured in a Varioskan Flash multi well reader (Thermo Scientific; Schwerte, Germany) at 660 nm. To check the data for normality the Shapiro-Wilk test was used. If the data was distributed normally, the significance of different proliferation rates was determined using a paired one-tailed T-test, if not a Mann-Whitney U test was performed.

## Results

### Expression of GARP variants in HEK 293H cells

**Fig 2:**
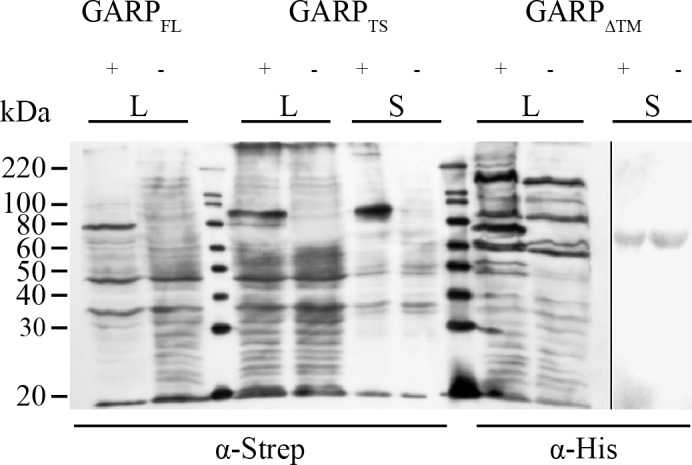
Transient Expression of the three recombinant GARP variants in HEK 293H cells. HEK 293H cells were transfected with plasmids containing the cDNA of the constructs GARP_FL_, GARP_TS_ and GARP_ΔTM_, respectively. 48h after transfection, the culture medium was exchanged for FCS-free DMEM supplemented with NEAA. Supernatants (S) and cell lysates (L) were obtained after another 48h of incubation. 1 ml of supernatant was precipitated using 2% (w/v) Na-deoxycholate solution (1:100) and 100% TCA (1:10). Cell lysates were prepared using 200 μl RIPA buffer per 1x10^6^ cells. Samples were separated on a 10% PAA SDS-PAGE followed by western blotting on a PVDF membrane. For molecular size determination the magic mark XP marker (Invitrogen; Darmstadt, Germany) was used. For detection the blot was probed with α-Strep-tag and α-His-tag antibodies, respectively (Quiagen; Hilden, Germany). As secondary antibody a peroxidase coupled anti-mouse-IgG antibody (Dianova; Hamburg, Germany) was used.

Starting from the full-length cDNA encoding human wild-type GARP (GARP_WT_), three recombinant variants were generated by PCR (Fig 1). The first one, GARP_FL_, comprises the complete sequence including a Strep-tag attached C-terminally of the transmembrane region. The second, GARP_TS_, is a tail switch mutant, in which the original transmembrane region was replaced with the C-terminal end of human meprin α (aa 606–746) and a preceding Strep-tag. Additionally, this construct contains a His-tag between the N-terminal signal peptide and the mature chain. In the third construct, GARP_ΔTM_, the trans membrane region was replaced by a His-tag. Heterologous expression of the three cloned GARP variants showed different properties with respect to their secretion. The final supernatant of the cells was isolated and the cells were harvested and lysed. As expected, GARP_FL_ resembled the positive control as shown in a single distinct signal, corresponding to a molecular mass of 74 kDa, in the immunoblot of the lysate of transfected HEK 293H cells (Fig 2). The expression of the tail switch mutant GARP_TS_ lead to secretion of the protein into the supernatant. The corresponding band shows a slightly increased molecular mass compared to GARP_FL_, due to the additional C-terminal meprin α moiety. Secretion is facilitated through an internal furin cleavage site in the meprin α tail, which is cleaved in the Golgi network and leads to loss of the transmembrane domain during secretion. In contrast, deletion of the transmembrane domain and addition of the His-tag (GARP_ΔTM_) did not result in secretion, but rather to protein accumulation in the cell, since a band at the molecular mass of 71 kDa was visible only in the cell lysates, but not in the supernatant (Fig 2).

### Expression and purification of GARP_TS_ in Hi5 insect cells

**Fig 3:**
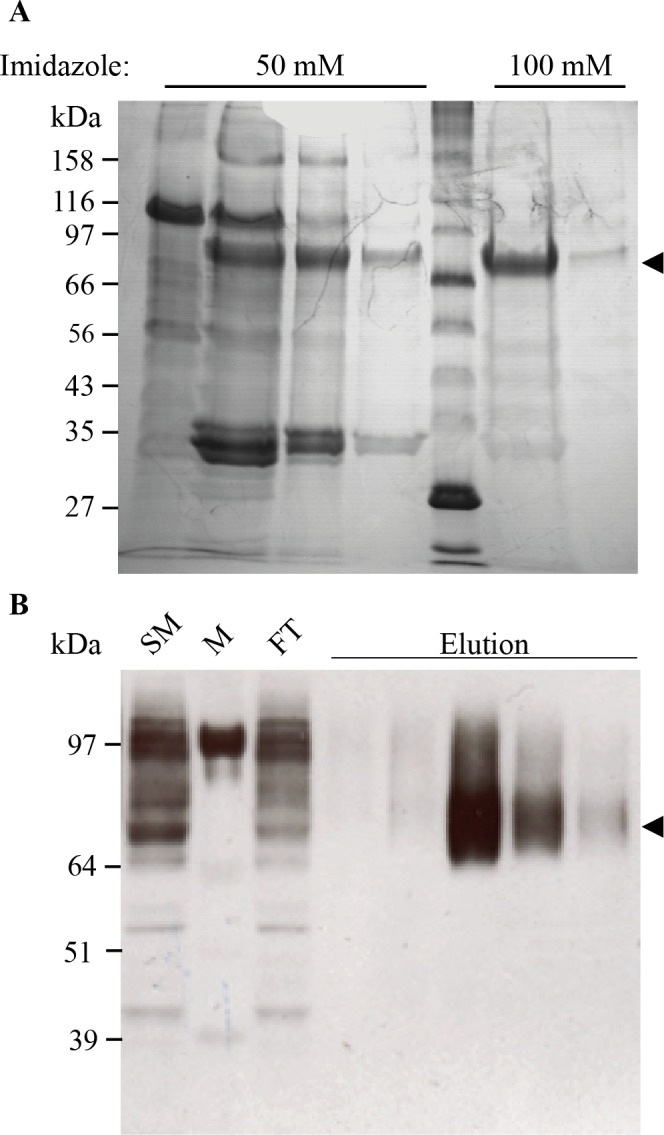
Electrophoretic analysis of GARP_TS_ fractions from the final Ni-NTA chromatography. (A) Elution fractions of the Affigel Blue column were pooled and proteins bound on the Ni-NTA matrix overnight. Lane 1–4: Washing fractions, containing 50 mM imidazole; Lane 5: Protein Marker Broad Range (NEB; Ipswich, USA); Lane 6+7: Elution fractions containing 100 mM imidazole. The arrowhead marks the soluble form of GARP_TS_ at a molecular weight of 75 kDa. Fractions were analyzed by 10% PAA SDS-PAGE und reducing conditions, followed by staining with coomassie brilliant blue. (B) The supernatant of TRP2_TS_ transfected Expi293F cells (Invitrogen; Darmstadt, Germany) were concentrated and applied to a Strep-Tactin matrix. SM: Starting Material; M: Protein Marker SeeBlue Plus 2 (Invitrogen; Darmstadt, Germany); FT: Flowthrough; Elution: Eluted fractions with 2.5 mM D-Desthiobiotin. The arrowhead marks the soluble TRP2_TS_ at a molecular weight of 75 kDa. Fractions were analyzed by 10% PAA SDS-PAGE und reducing conditions followed by western blotting on a PVDF membrane. For detection the blot was probed with anti-TRP2 antibody (Abcam; Cambridge, UK). As secondary antibody a peroxidase coupled anti-rabbit-IgG antibody (GE Healthcare; Solingen, Germany) was used.

It was possible to produce soluble GARP_TS_ at a larger scale by using an expression system based on the infection of Hi5 insect cells with recombinant baculoviruses carrying the GARP_TS_ cDNA. After 4 days of incubation the conditioned media was collected and cells removed via centrifugation. GARP_TS_ was purified using a tandem-chromatography strategy consisting of an Affi-Gel Blue column (Biorad Munich, Germany) and in a second step a Ni-NTA column. GARP_TS_ could be eluted from the Affi-Gel Blue using 2M NaCl, was then dialyzed and loaded on the Ni-NTA column, from which a homogeneous single band corresponding to GARP_TS_ was desorbed at an imidazole concentration of 100 mM (Fig 3A). The yield was about 0.9 mg of GARP_TS_ from one liter of conditioned media. For the following assays and long term storage, GARP_TS_ was dialyzed against 150 mM NaCl, 20 mM Tris/HCl, pH 8.0, 5 mM CaCl_2_, 0.05% (w/v) Brij-35 and stored frozen at -20°C.

To proof the concept of using the meprin α tail for expression and solubilization of other membrane bound proteins, we also tested this strategy for the cancer related tyrosinase TRP-2 [26] (UniProtKB P40126, TRP2_HUMAN, tyrosinase related protein 2, DOPAchrome tautomerase). This construct was transfected in HEK 293 Expi cells (Invitrogen; Darmstadt, Germany). The supernatant of these cells was taken 48 h after transfection and the recombinant TRP-2 tails witch mutant was purified using Strep-tactin sepharose. Different steps of the purification were analyzed through immunodetection using a TRP-2 specific antibody (Fig 3B), demonstrating that almost all recombinant TRP-2 bound to the Strep-tactin column and could be eluted after the addition of 2.5 mM D-desthiobiotin (Fig 3B).

### Recombinant GARP_TS_ binds TGFβ and enhances the growth factor’s suppressive activity

**Fig 4:**
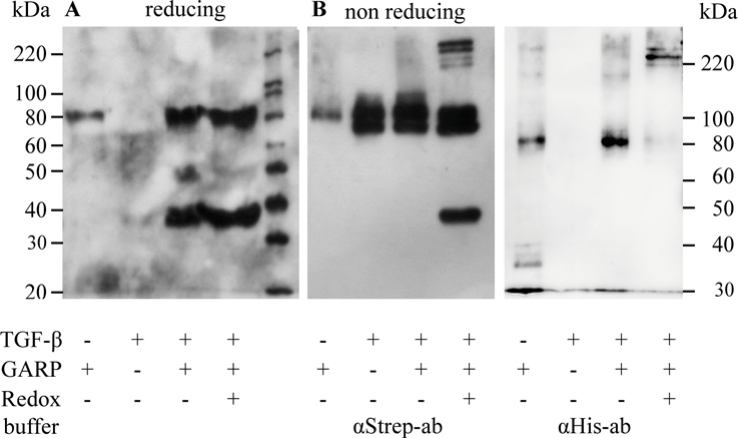
*In vitro* coupling of GARP_TS_ to recombinant TGFβ. (A) 300 ng GARP_TS_ and 600 ng recombinant latent TGFβ per lane were incubated overnight in the presence or absence of a redox-buffer containing 0.05 mM GSSH and 2 mM free cysteine at RT to allow the formation of GARP-LAP interactions. Then the samples were incubated for 4 hours at 4°C together with magnetic Ni-NTA beads to bind GARP_TS_ and putative GARP-LAP complexes at the beads. Proteins bound to the beads were analyzed by western blot with anti-Strep-tag antibodies, which could be used to detect both GARP_TS_ and recombinant TGFβ. (B) Equally treated controls were analyzed on a 10% PAA SDS-PAGE under non-reducing conditions but without the pull-down procedure. After gel electrophoresis, proteins were blotted onto a PVDF membrane and probed with anti-Strep-tag (left) or anti-His-tag antibodies (right), respectively.

*In vivo* GARP binds TGFβ covalently via disulfide-bonds, which are formed during post-translational processing in the ER-Golgi network and the GARP-bound TGFβ exhibits enhanced activation. We first tested whether the soluble tail-switch mutant GARP_TS_ was able to bind latent TGFβ. Therefore, GARP_TS_ was incubated with latent TGFβ and a pull-down assay demonstrated that latent TGFβ was indeed bound by soluble recombinant GARP_TS_ (Fig 4A). Pull-down experiments were performed both in the absence and in the presence of oxidized glutathione and free cysteine in order to clarify, whether covalent bonding was involved in this interaction. In both settings, with or without redox-system, we observed binding of GARP_TS_ to latent TGFβ.

Moreover, as shown for GARP_TS_ samples pretreated with a mixture (double band) of pro-TGFβ and latent TGFβ (i.e. furin-cleaved pro-TGFβ), the direct formation of the disulfide-bridged states could be observed by non-reducing SDS-PAGE, as visualized by western-blotting and subsequent detection using anti-Strep-tag or anti-His-tag antibodies, respectively (Fig 4B). Anti-Strep-tag antibodies detect both recombinant TGFβ and GARP_TS_, whereas anti-His-tag antibodies only detect GARP_TS_ (compare Fig 1). In the glutathione treated sample two double bands appeared at positions corresponding to molecular masses of approximately 170–190 kDa and 230–250 kDa (Fig 4B left panel; detection with anti-Strep-tag-antibodies). This indicates the ability of GARP to bind both pro-TGFβ and latent TGFβ. Moreover, the molecular size of the two double bands suggests a stoichiometry of two molecules of GARP binding one molecule of TGFβ (double band of 230/250 kDa) and in addition a 1:1 stoichiometry (less abundant double band of 170/190 kDa). Incubation of 300 ng GARP_TS_ and 600 ng TGFβ in the aforementioned redox-buffer is sufficient to completely convert GARP_TS_ to the high molecular GARP-TGFβ complex (see Fig 4B, right panel; detection with anti-His-antibodies).

**Fig 5:**
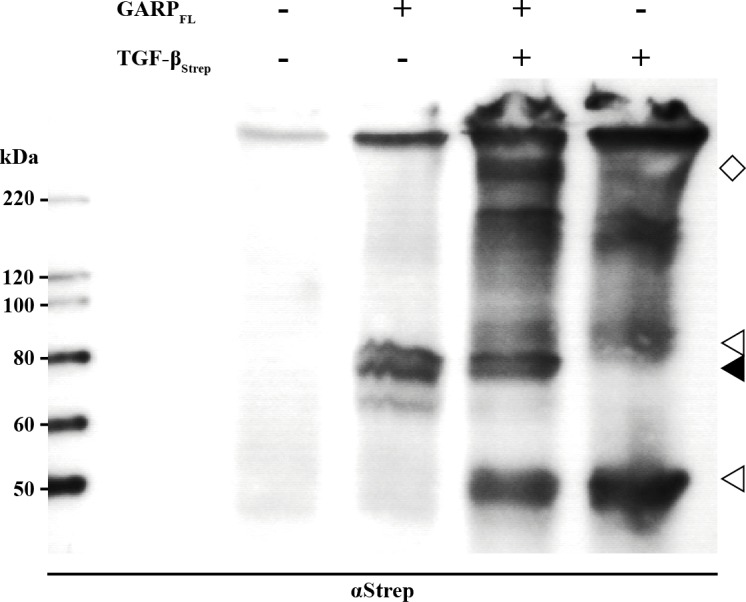
*In vivo* coupling of GARP_TS_ to recombinant TGFβ. HEK 293H cells were transfected with plasmids containing the cDNA of the constructs GARP_FL_, TGFβ_Strep_ or both in combination. 48h after transfection, the culture medium was exchanged for FCS-free DMEM supplemented with NEAA. Cell lysates were prepared using 200 μl RIPA buffer per 1x10^6^ cells. Samples were separated on a 10% PAA SDS-PAGE followed by western blotting on a PVDF membrane. For molecular size determination the magic mark XP marker (Invitrogen; Darmstadt, Germany) was used. For detection the blot was probed with anti-Strep-tag and anti-His-tag antibodies, respectively (Quiagen; Hilden, Germany). As secondary antibody a peroxidase coupled anti-mouse-IgG antibody (Dianova; Hamburg, Germany) was used.

To verify the observed complexes of GARP_TS_ and latent TGFβ in an *in vivo* like situation HEK 293H cells were transfected with plasmids containing the cDNA for a tagged version of latent TGFβ and a tagged version of full-length GARP alone or in combination. Four days after transfection cells were harvested and then lysed in RIPA-buffer. Anti-Strep-tag antibodies detected both GARP_FL_ and TGFβ_Strep_ subsequent to non-reducing SDS-gelelectrophoresis (Fig 5). Transfection with GARP_FL_ alone resulted in a band at 80 kDa (black arrowhead). Full-length monomeric TGFβ (45 kDa) and a weaker double band of pro- and latent TGFβ (90 kDa) are indicated by a white arrowhead. The white diamond marks the prominent complex of co-transfected TGFβ and GARP. This signal appears at the same molecular size of 240 kDa as in the *in vitro* experiments of GARP_TS_ and TGFβ coupling (Fig 4), suggesting the same molecular ratio of TGFβ and GARP *in vivo*.

**Fig 6:**
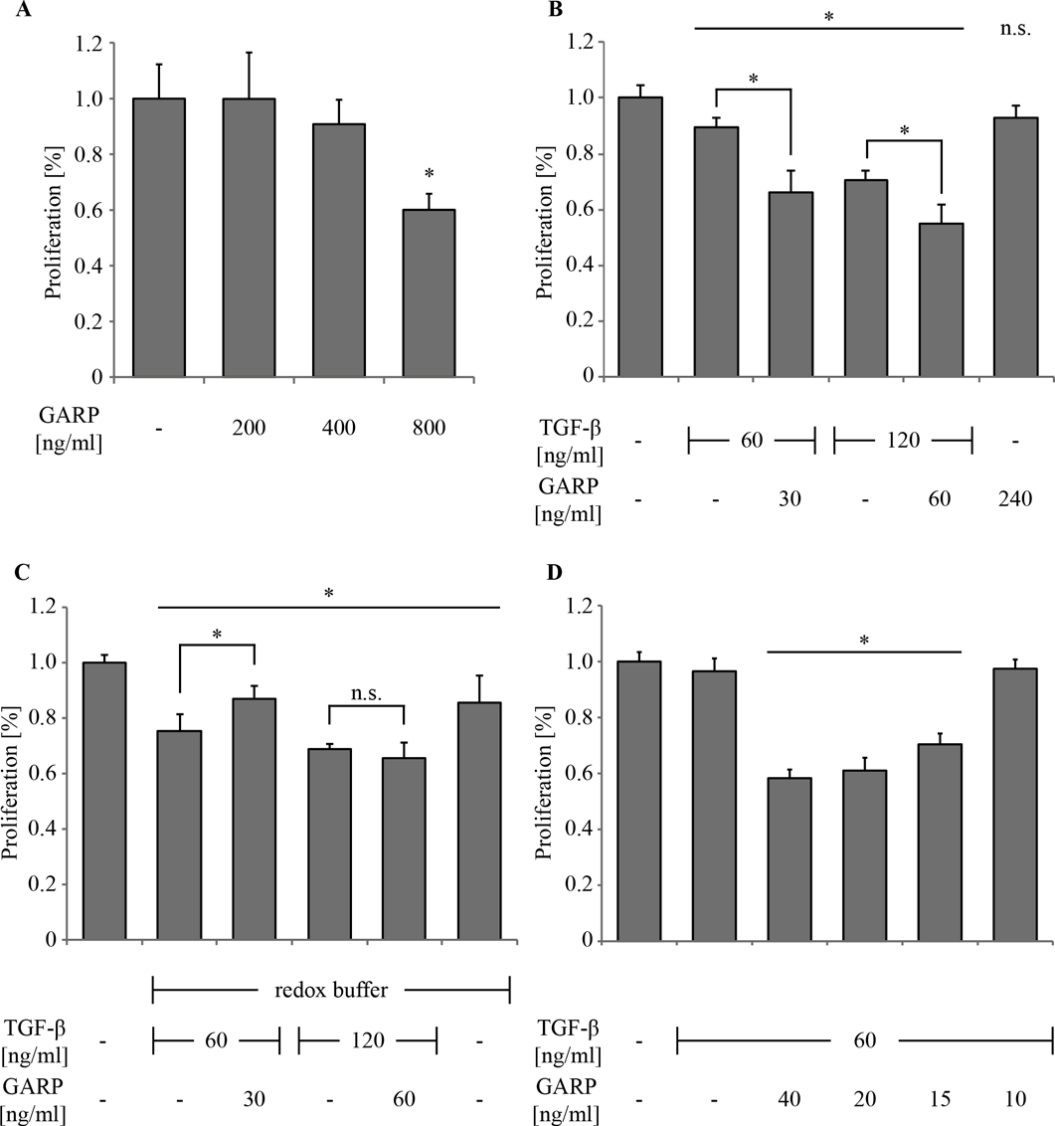
Inhibition of cell proliferation of Mv1Lu cells by GARP and TGFβ. Bar diagrams show the proliferation rates (percentage of proliferation, n = 6) of treated versus untreated (100%) Mv1Lu cells. Cell numbers were determined using methylene blue staining of DNA [25]. (A) 2.5 x 10^4^ cells were grown for 48h and treated with 200 ng/ml to 800 ng/ml of recombinant GARP_TS_ to determine its influence on cell proliferation alone. (B) Cells were grown for 48h in the presence of the indicated TGFβ concentrations pre-incubated with or without the indicated GARP_TS_ concentration. (C) Cells were incubated the same way like in (B), except that the proteins were pre-incubated in redox buffer to provoke covalent bonding of TGFβ and GARP (D) 60 ng/ml TGFβ were pre-incubated with different amounts of GARP_TS_ (40–10 ng/ml) to determine the minimal amount of GARP_TS_ needed to achieve maximal enhancement of TGFβ activation. The asterisk (*) marks a statistical significance p < 0.01.

Different publications show that the application of soluble GARP can modulate the immune response for example by inducing IL-2 or by reducing IFN-γ [24]. However, the underlying mechanisms are still obscure. It has been assumed that membrane association and disulfide coupling between GARP and TGFβ might be prerequisites for correct GARP functionality [21]. Since we showed the ability of soluble GARP_TS_ to bind TGFβ either non-covalently or via disulfide-bridges, we examined the impact of non-covalent and covalent binding with respect to the activability of TGFβ. Therefore, a selective, well established assay for the anti-proliferative effect of active TGFβ was employed, based on the cytokine’s ability to drive mink (Mv1Lu) cells into cell cycle arrest in the G1/GO phase via SMAD signaling [27]. These cells were as sensitive to GARP_TS_ alone as CD4^+^ T cells. While at a concentration of 200 ng/ml no alteration in cell proliferation was visible, a concentration of 400 ng/ml caused a reduction of cell proliferation, which was even more significant at 800 ng/ml GARP_TS_ (Fig 6A). Application of latent TGFβ (60 and 120 ng/ml) alone caused only a slight decrease of cell proliferation to 90% and 70%, respectively (Fig 6B). Proliferation was further decreased by the addition of GARP_TS_ to 65% and 55%, respectively, upon co-incubation of TGFβ and GARP_TS_ in the absence of a redox buffer and thus only non-covalently connected. This shows the principal ability of GARP to enhance the intrinsic TGFβ activation by its ability to bind the growth factor. By contrast, no enhancement could be observed beyond the level of proliferation caused by latent TGFβ alone, when latent TGFβ was covalently coupled to GARP_TS_ in redox buffer (Fig 6C). In fact, at a concentration of 60 ng/ml TGFβ and 30 ng/ml GARP_TS_ a slight neutralizing effect caused by GARP_TS_ was observed, which was not seen at 120 ng/ml TGFβ and 60 ng/ml GARP_TS_, respectively. In general, the redox buffer by itself seems to slightly inhibit the proliferation of Mv1Lu cells, which might explain the lower levels of proliferation compared to Fig 6B. Titration of a constant amount of latent TGFβ (60 ng/ml) with increasing concentrations of GARP_TS_ resulted in a proportional decrease of cell proliferation (Fig 6D). Treatment of the cells with latent TGFβ alone resulted only in slightly decreased proliferation. However, upon addition of GARP, the proliferation was significantly decreased to below 60% at a concentration of approximately 40 ng/ml (0.5 nM). Hence, GARP_TS_ seems to enhance latent TGFβ activation only, if it is bound non-covalently.

### Binding of GARP to TGFβ induces a conformational change

**Fig 7:**
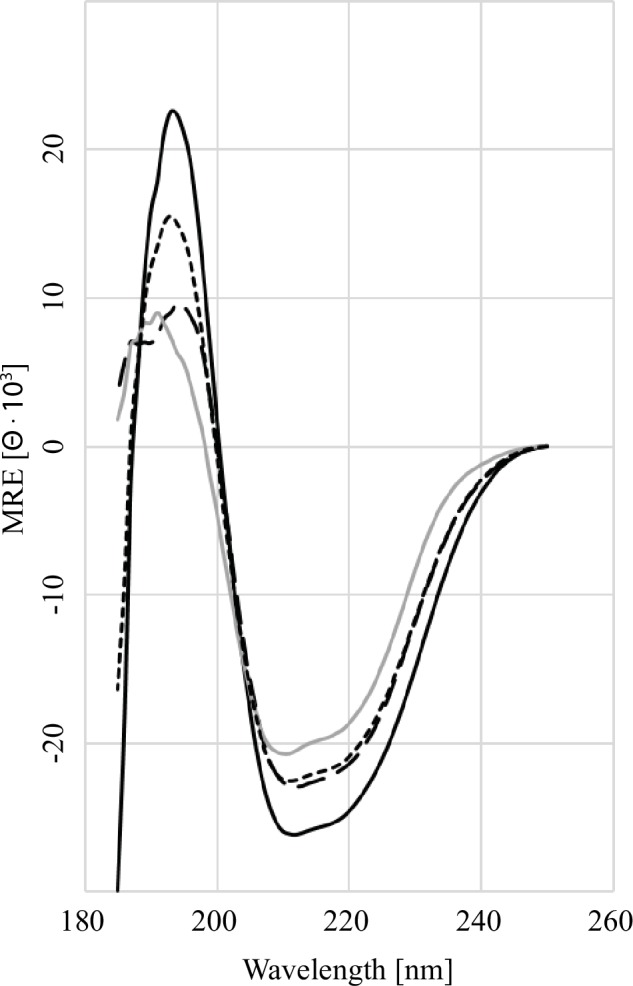
Far UV CD-Spectroscopy of GARP and TGFβ. The far-UV spectra (25°C, 260–185 nm) for the following samples are as indicated: GARP_TS_ alone (black solid line), latent TGFβ alone (grey solid line), summed spectrum of GARP_TS_ and latent TGFβ (short-dashed line), measured spectra of the GARP_TS_ and latent TGFβ complex (long dashed line). The decrease in the mean relative ellipticity below 200 nm between the calculated and measured spectra of the complex formation of GARP_TS_ and TGFβ indicates a conformational change in GARP.

In order to analyze the molecular arrangement of the GARP-TGFβ complex far UV spectra of GARP_TS_ TGFβ and the non-covalent complex of both proteins were recorded (Fig 7). Due to the high absorbance of the buffer it was possible to obtain a clear CD-spectrum only above 185 nm. The CD spectrum of TGFβ is comparable to previously published ones [28] indicating correct folding (grey solid line). The spectrum of GARP_TS_ exhibits a minimum at 212 nm, a shoulder at 217 and a maximum at 193 nm. In addition, there is a strong drift into negative elliptical values below 188 nm. This spectrum is consistent with a high amount of helically ordered β-strands, which are typical for LRR containing proteins as confirmed for GARP by electron microscopy and molecular modeling [10]. To investigate whether there are conformational changes upon latent TGFβ binding, GARP_TS_ and the recombinant latent TGFβ dimer were mixed at a molar ratio of 2:1. The obtained spectrum (black long dashes) was compared with the theoretical spectra calculated for the individual amino acid sequences of GARP_TS_ and latent TGFβ (black small dashes). In the region of 200 nm to 250 nm no significant differences between the measured and calculated spectra could be observed. But at wavelengths below 200 nm the spectra diverge. The calculated maximum at 192 nm is decreased by 6000 units and shifted towards a longer wavelength of 195 nm compared to the measured one. This indicates a conformational rearrangement in the helical, overall structure of GARP_TS_ upon TGFβ binding.

## Discussion

Various strategies have been developed for the recombinant expression of soluble GARP, either by omitting the transmembrane anchor [21] or by replacing it with the Fc-domain of immunoglobulin G [24]. We decided to mimic soluble GARP as generated *in vivo* by proteolytic ectodomain shedding from T cells [23]. To ensure proper post-translational modification and secretion, we replaced the C-terminal segment of GARP, including its transmembrane anchor, by the C-terminal part of the human metalloproteinase meprin α. We had carried out a similar experiment for the sister subunit meprin β, which in contrast to meprin α is translocated to the plasma membrane as a typical type I ectoprotein [29]. The subtle difference in meprin α is the presence of a furin cleavage site amino-terminally of its transmembrane domain. The meprin α tail switch offers significant advantages compared to other tags like the Fc-tag, since it implicates only minimal alterations in the structure of the target protein. Another advantage of this approach is its versatility and broad applicability to a variety of commonly used eukaryotic expression systems like HEK 293 cells, CHO cells, SF9 cells and Hi5 cells, which all contain the enzymatic repertoire capable of cleaving furin sites. Proof of principle was demonstrated with a completely different membrane protein, namely the ‘tyrosinase related protein 2’.

After confirming that GARP was able to bind TGFβ *in vitro* it was possible to demonstrate that soluble GARP can enhance the activation of TGFβ. Most interestingly, this effect was only seen when latent TGFβ was coupled non-covalently to GARP. This is in accordance with published work by Springer and co-workers [21], who showed that soluble GARP did not exhibit TGFβ-enhancing potency, if it was covalently complexed with the small latent LAP-TGFβ, which occurs co-translationally in the oxidizing environment of the endoplasmic reticulum. In this covalent soluble GARP-TGFβ complex, the mechanical force required to release active TGFβ via target cell-integrins [30] cannot come into effect, because GARP is not tethered to its mother cell [21]. The two requirements postulated by Springer and co-workers [21], GARP has to be membrane bound and TGFβ coupled by disulfide bridges, can be extended by our findings. If TGFβ free GARP is released from the cell surface, it can bind to latent TGFβ non-covalently and enhance its activation. However, enhancement is impossible if TGFβ is bound covalently.

In another study, T cells were incubated with soluble GARP at concentrations of up to 1 μg/ml, which resulted in a significant up-regulation of TGFβ and a concomitant decrease of cell proliferation and cytokine expression [24]. Whether these amounts of GARP reflect the *in vivo* situation remains open. However, this anti-proliferative effect of GARP at a high dose could be confirmed in our studies. GARP at (11 nM) 0.8 μg/ml was able to suppress cell proliferation of Mv1Lu cells down to 60%, whereas (5 nM) 0.4 μg/ml did not show a significant effect. All these observations support the hypothesis that soluble GARP can bind free latent TGFβ non-covalently and thereby enhance its activation. In this scenario even small amounts of free latent TGFβ can be shanghaied and activated by soluble GARP. Possibly this would result in a positive feedback by up-regulating TGFβ expression, which again can bind to soluble GARP. Such a positive feedback loop has been described for GARP and FoxP3, claiming GARP to act as a safeguard for the regulatory phenotype of Treg [31]. It seems there is another mediator of this feedback loop, namely latent TGFβ, which is activated by GARP to stimulate its own expression as well as the expression of Foxp3. The ability of GARP to take up exogenous TGFβ and to enhance its activation reveals a way how other cells might influence immunologic tolerance. In fact, certain cancer cells are known to express TGFβ and GARP by themselves.

Questions remain, e.g. regarding the stoichiometry of the GARP:LAP-TGFβ interaction. Previous mutagenesis studies had indicated GARP’s Cys192 and Cys331 as potential interaction sites for latent TGFβ binding, suggesting a 1:1 stoichiometry for GARP:LAP-TGFβ [21]. The data of the present study rather suggest a 2:1 stoichiometry for GARP:LAP-TGFβ. This is supported by non-reducing western blot analysis, which shows a band corresponding to a relative mass of 250 kDa indicating a disulfide bridged complex of a single LAP-TGFβ dimer bound by two molecules of GARP. Homology modeling of GARP revealed only a single free cysteine (Cys345) in the mature chain, which could be responsible for the binding to Cys4 of LAP-TGFβ. Interestingly, in the proliferation assay a stoichiometry of 2:1 for GARP:LAP-TGFβ was not required to achieve maximal enhancement of TGFβ activity. It was rather a ratio of 1:2, that caused maximal anti-proliferative effect even at concentrations of 0.5 nM of GARP. This might be due to fact that the assembly of the GARP-LAP-TGFβ complex is not the rate-determining step in TGFβ activation, because of the high affinity of GARP towards LAP-TGFβ. Other events like the disruption of the latent LAP-TGFβ complex might be much slower and restricted through the concentration of the activator. Another explanation for these findings could be that GARP is not only involved in TGFβ activation, but rather binds to its receptors and thereby prolongs the half-life of the receptor-ligand complex on the cell surface, which would lead to an increased signal. Hahn et al. (2013) [24] showed an anti-immunogenic effect of GARP alone at high concentrations on *ex vivo* T cells. This observation supports our hypothesis of soluble GARP being able to bind exogenous TGFβ and enhance its activation, since T cells, once activated express latent TGFβ at high amounts on their own. Nevertheless, the CD-spectral analyses support the hypothesis that concomitant binding of latent TGFβ and GARP induce a conformational change, which leads to the exposition of regions (such as the RGD motif) which are necessary for TGFβ activation.

The biological function of the two binding mechanisms remains intriguing. *In vivo*, endogenous TGFβ is only found disulfide-linked to GARP on the surface of Treg. In order to bind to GARP, latent TGFβ has to attach first to the receptor before it can be disulfide-linked. The fact that GARP outcompetes other TGFβ binding proteins like LTBP and that nearly all TGFβ could be recovered in pull down assays suggests a very strong affinity of GARP towards pro and latent TGFβ. Under extracellular conditions such a mechanism could play a role in the uptake and activation of exogenous TGFβ.

An even more striking result of this study is the finding that soluble GARP can be used in combination with TGFβ, to enhance the activation process of this cytokine. Taken together with the findings from Hahn et al. (2013) [24] this opens further strategies to use GARP as immune modulatory agent. In settings where immune suppression might be beneficial, e.g. transplantation or autoimmune disease, the simultaneous administration of soluble GARP and latent TGFβ could be a promising attempt. This method would offer two advantages. Firstly, the required amount of recombinant protein (120 ng/ml TGFβ and 60 ng/ml GARP) would be much lower than with GARP alone (1 μg/ml GARP) [24], which makes this setting much more feasible to realize in translational studies or later in a clinical approach. Secondly, compared with the usage of pre-activated TGFβ, GARP can bind the latent form of TGFβ and enhance its activation specifically, since integrins are necessary for the final activation step [21]. This would minimize the risk of putative side effects of exaggerated TGFβ activation.
